# Maternal Adherence to the Mediterranean Diet and Adverse Pregnancy Outcomes: Findings from the Multi-Center PLATONE Project

**DOI:** 10.3390/nu18050769

**Published:** 2026-02-26

**Authors:** Simona Esposito, Sabatino Orlandi, Teresa Panzera, Paola De Domenico, Carmine Malzoni, Pietro Mastandrea, Francesca De Micco, Augusto Di Castelnuovo, Chiara Cerletti, Marialaura Bonaccio, Licia Iacoviello

**Affiliations:** 1 Research Unit of Epidemiology and Prevention, IRCCS Neuromed, Via dell’Elettronica, 86077 Pozzilli, IS, Italy; 2 Department of Medicine and Surgery, LUM University, 70010 Casamassima, BA, Italy; 3 Istituto Clinico Mediterraneo, 84043 Agropoli, SA, Italy; 4 Malzoni Research Hospital, 83100 Avellino, AV, Italy; 5 Hyppocratica Spa Casa di Cura Villa del Sole Sede Operativa via dei Greci, 84135 Fratte, SA, Italy; 6 Mediterranea Cardiocentro, 80122 Napoli, NA, Italy

**Keywords:** Mediterranean Diet, pregnancy complications, cesarean delivery, stillbirth outcomes

## Abstract

**Background/Objectives:** Pregnancy is a critical period during which lifestyle factors, including diet, can affect maternal and fetal outcomes. The Mediterranean Diet (MD) may offer advantages, but evidence on its impact on adverse pregnancy outcomes is limited. We evaluated the association between an MD and adverse pregnancy outcomes, cesarean delivery, and stillbirth outcomes in a large sample of hospitalized women in Italy. **Methods:** A cross-sectional analysis of retrospectively assessed dietary exposure was conducted using data from 1511 pregnant women recruited within the multi-center PLATONE project. Adherence to the MD was assessed through the nine-item MEDI-LITE. Multivariable logistic regression was used to evaluate the relationship between MD and adverse pregnancy outcomes. **Results:** High, average, and poor adherence to the MD were reported by 30.8%, 45.3%, and 23.9% of participants, respectively. In a multivariable adjusted analysis, each one-unit increase in the MEDI-LITE adherence was associated with a lower likelihood of adverse pregnancy outcomes (OR = 0.94; 95%CI: 0.88–0.99), while downward trends were also observed for cesarean delivery (OR = 0.96; 0.90–1.01) and stillbirth (OR = 0.94; 0.83–1.06). Among specific MEDI-LITE components, moderate intake of dairy products was associated with reduced adverse pregnancy outcomes, while moderate-to-high fruit consumption was linked to lowered cesarean odds. **Conclusions:** Maternal adherence to an MD was associated with lower odds of adverse pregnancy outcomes. Given the observational design and because MEDI-LITE is a validated adherence score rather than a tool to test causal effects on pregnancy outcomes, these findings should be interpreted as associations and do not allow causal inference.

## 1. Introduction

During pregnancy, a woman’s body undergoes a series of significant physiological changes to adapt and accommodate the development of the growing fetus [1]. For this reason, it is crucial to pay close attention to the maternal diet during this highly sensitive period [2]. A major public health problem is the increased prevalence rates of preeclampsia, gestational diabetes mellitus, and excessive gestational weight gain during pregnancy that reflect the increasing prevalence of obesity and changes in women’s lifestyles [3,4,5].

A growing number of studies showed the protective effects of a Mediterranean Diet (MD) on maternal outcomes and children’s health [6,7,8]. The traditional MD is characterized by daily intakes of vegetables, fruits, cereals, high intake of nuts and legumes, moderate intakes of dairy products, fish, and wine during main meals, and low intakes of red meat and processed meat, and olive oil as the main source of added fat [9]. This dietary pattern has been extensively explored for its high polyphenol content, with anti-inflammatory and antioxidant properties [10,11]. Indeed, the MD is suggested to exert a protective effect against gestational diabetes mellitus, potentially mediated by a high intake of polyphenols found in key dietary components such as extra virgin olive oil, fruits, and vegetables. Dietary polyphenols may enhance insulin sensitivity, reduce glycemic load, activate insulin receptors, stimulate insulin secretion, and modulate glucose release, ultimately promoting glucose uptake in insulin-sensitive tissues and contributing to the control of excessive gestational weight gain [12]. Moreover, a recent trial reported inverse associations in relation to an early nutritional intervention with a supplemented MD and gestational diabetes mellitus [13]. On the contrary, high consumption of potatoes, meat, processed meats, and animal protein was associated with an increased risk of gestational diabetes mellitus [14]. Globally, preeclampsia, defined as the emergence of hypertension after the 20th week of pregnancy coupled with proteinuria and/or maternal complications, as well as fetal growth restriction [15], affects 2–8% of pregnancies and is one of the major contributors to both maternal and fetal complications [16].

A recent review reported that lifestyle factors, such as regular exercise, stress reduction, and a healthy diet, play a significant role in preventing preeclampsia and promoting overall maternal well-being [17].

While the protective effects of an MD on common maternal outcomes are well documented, evidence on its impact on delivery outcomes, such as the risk of cesarean section, and on fetal outcomes, including stillbirth, remains limited [8,13].

Moreover, to the best of our knowledge, a few studies have been conducted in Southern Italy, a region experiencing a nutrition transition, characterized by declining adherence to a traditional MD [18], despite its historical role as the birthplace of this dietary pattern.

To fill this knowledge gap, our study aimed to explore the association of an MD with adverse events during pregnancy, including the risk of cesarean delivery or stillbirth outcomes.

## 2. Materials and Methods

### 2.1. Study Population

Pregnant women (*n* = 2511) were recruited from March 2019 to May 2023 from four clinical centers of the Neuromed Clinical Research Network in Central and Southern Italy, within the framework of the Big Data and Personalized Health Project [19]. Further details about the network are available in [19]. Inclusion criteria were being hospitalized for at least 24 h for childbirth and/or pregnancy complications in the following clinics of the Neuromed Network, which housed an obstetrics and gynecology department: Clinica Mediterranea (Napoli), Istituto Clinico Mediterraneo (Agropoli, Salerno), Villa del Sole (Salerno), Malzoni Research Hospital (Avellino). Exclusion criteria were hospitalization for day surgery, intensive care unit admission, and age under 18 years. The study was conducted in accordance with the Declaration of Helsinki and approved by the Ethical Committees of IRCCS Neuromed and all participating clinical centers. All participants provided written informed consent, permitting the collection of personal and clinical information, the retrieval and storage of residual biological samples in a biobank for future omics analyses, and the conduction of follow-up to monitor their health status. Upon consent, patients were given questionnaires to complete during their hospital stay, which were then returned to the local research monitor (one or two per recruiting center). Among all participants, women completed the dietary questionnaire during the first days of the hospital stay that ended with delivery. A very small number (*n* = 43; 2.8%) completed it during an earlier hospitalization for check-ups, giving birth a few months later. The study can be considered virtually cross-sectional, since exposure was measured at approximately the same time as the outcome.

### 2.2. Data Collection

Adherence to the MD was evaluated during hospitalization using the MEDI-LITE questionnaire [20], completed in the first days of the hospital stay and referring to habitual dietary habits in the year preceding enrolment. The MEDI-LITE assesses the consumption of nine food groups and classifies dietary patterns based on MD principles. It was chosen because it was specifically developed and validated for the Italian population and is suitable for use in observational epidemiological studies.

For food groups typical of the MD (i.e., fruit, vegetables, cereals, legumes, and fish), scores were assigned as follows: 2 points for the highest consumption category, 1 point for the intermediate category, and 0 points for the lowest category. For food groups not characteristic of the MD (i.e., meat and meat products and dairy), the scoring was inverted: 2 points for the lowest consumption, 1 point for intermediate consumption, and 0 points for the highest consumption. Alcohol intake was scored according to alcohol units (1 unit = 12 g of alcohol): 2 points for moderate consumption (1–2 units/day), 1 point for low consumption (≤1 unit/day), and 0 points for high consumption. Finally, olive oil use was scored as 2 points for regular use, 1 for frequent use, and 0 for occasional use. The total score ranges from 0 to 18, with higher scores indicating greater adherence to the MD. For analytical purposes, the score was categorized into tertiles: poor (0–9 points), average (10–11 points), and high (12–17 points) adherence. Patients were asked to provide, through self-administered questionnaires, additional lifestyle information, e.g., about the type of water they drink (tap, in plastic or in glass bottle), smoking status (ever, past and current smokers, no of cigarettes per day and years of smoking), level of physical activity both in leisure time and during working hours, and habits of use with regard to cordless or mobile phones (no of hours per day). Sleep quality and duration were self-reported, including whether sleep was continuous or interrupted, and whether participants worked night shifts, along with the frequency of such shifts per week, if applicable. Socioeconomic status was assessed by collecting information on marital status (i.e., married/living in a couple, separated/divorced, single, widower), educational level (i.e., lower, upper secondary, and postsecondary education), and occupational category (i.e., student, manual, non-manual, professional and managerial worker, housewife, retired, unemployed). Prevalent clinical conditions such as hypertension, diabetes, and hypercholesterolemia were defined based on participants’ reports of current pharmacological treatments for one or more of these conditions. Height and weight were self-reported, and body mass index (BMI) was calculated as kg/m^2^ and then grouped into three categories: under/normal weight (<25), overweight (≥25 to <30), or obese (≥30). Biometric data, including systolic and diastolic blood pressure and heart rate, were measured by physicians at the clinics, and information was collected from patients’ medical records. The number of pregnancies during the course of life was also collected.

### 2.3. Outcome Assessment

Personal information, hospitalization causes, and final diagnoses were retrieved monthly from a dedicated digital platform for health data management and integration system. This centralized platform integrates biomedical and clinical data from all patients admitted to hospitals participating in the collaboration. The main adverse pregnancy outcomes investigated were: (a) previous cesarean delivery complicating pregnancy, labor, and the puerperium; (b) ballotable fetal head at term; (c) prolonged pregnancy; (d) oligohydramnios; (e) breech presentation without mention of external cephalic version; and (f) premature rupture of membranes. These outcomes were defined based on clinical information, hospitalization causes, and final diagnoses, and were classified into three categories according to their predominant association with pregnancy, delivery, or the fetus (see Supplementary Table S1).

Hospitalization causes and final diagnoses (type of delivery, live birth or stillbirth) were identified through ICD-9 codes within the ranges 630–677 and V27.

### 2.4. Statistical Analysis

For the present analysis, we excluded pregnant women who did not complete the questionnaire (*n* = 816) or who lacked data on pregnancy and birth outcomes (*n* = 181). The final sample size available for analysis was 1511 patients (Supplementary Figure S1). There were no significant differences in age between the included and excluded participants (33.1 ± 5.2 vs. 33.1 ± 5.5 years, respectively).

Characteristics of the study population were presented as numbers and percentages, or mean values and standard deviation (±SD) for continuous variables. Multivariable logistic regression analyses were used to estimate the association between the MEDI-LITE score (independent variable, modeled either as a continuous or categorical independent variable) or its individual dietary components (treated as a three-level variable) with pregnancy and birth outcomes (dependent variable), and results were expressed as odds ratios (ORs) with 95% confidence interval (95% CI). Covariates were selected based on evidence from existing literature [21]. Model 1 was adjusted for age only, while Model 2 included additional adjustments for age, occupation, marital status, educational level, leisure-time physical activity, smoking habit, BMI, number of previous pregnancies, hypertension, diabetes, and hyperlipidemia. Missing values for covariates, i.e., educational level (*n* = 25), smoking status (*n* = 1), leisure-time physical activity (*n* = 25), BMI (*n* = 13), diabetes (*n* = 10), hypertension (*n* = 5), hyperlipidemia (*n* = 5), marital status (*n* = 7), and occupation (*n* = 44), were handled using a multiple imputation technique (SAS PROC MI, *n* = 10 imputed datasets, followed by PROC MIANALYZE) to maximize data availability for all variables, avoid bias introduced by not-at-random missing data patterns and achieve robust results over different simulations. Data analyses were generated using SAS/STAT software, version 9.4 (SAS Institute Inc., Cary, NC, USA).

## 3. Results

**Figure 1:**
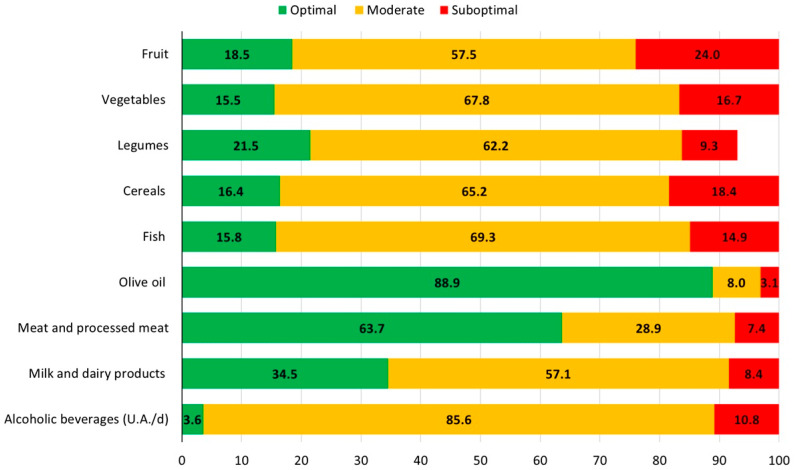
Proportion of participants with optimal, moderate, and suboptimal consumption of each food group in the MEDI-LITE questionnaire.

A total of 1511 (mean age 33.1 ± 5.2 years) were finally analyzed for the study. The majority of participants were married or living in a couple (87.2%), held an upper secondary education title (50.7%), were never smokers (65.4%), and were physically active (57.3%) (Table 1). Low, medium, and high adherence to the MD were reported by 23.9%, 45.3%, and 30.8% of study participants, respectively. Olive oil and meat/processed meat showed the highest proportion of participants with optimal consumption (88.9% and 63.7%, respectively). In contrast, alcohol, vegetables, cereals, and fish had a majority of participants in the moderate consumption category, with only 11–18% achieving optimal intake, highlighting potential areas for dietary improvement. Suboptimal consumption was most pronounced for fruit (24%), cereals (18.4%), and vegetables (16.7%) (Figure 1).

Compared with women with low MD adherence, participants with higher adherence were older, tended to be well educated, were mostly employed in non-manual work, were never smokers, practiced more physical exercise, and tended to have less hyperlipidemia (Table 1). During this study, a total of 873 cases of adverse pregnancy outcomes (i.e., pregnancy complications = 195; delivery complications = 787; fetal complications = 56), 747 cesarean deliveries, and 83 stillbirth cases were documented.

**Figure 2:**
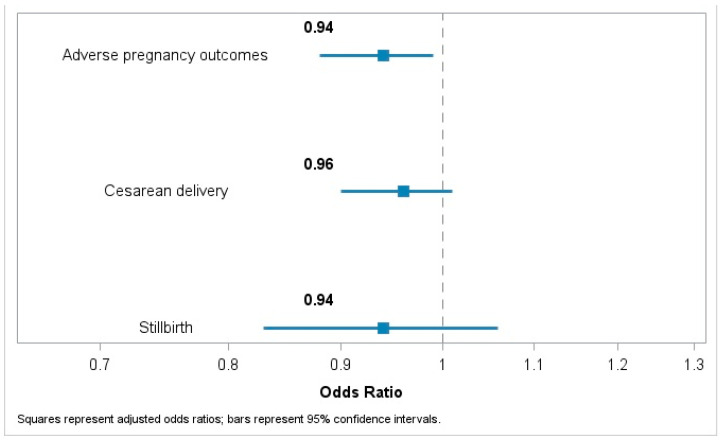
Association of a one-unit increase in the MEDI-LITE score with adverse pregnancy outcomes, type of delivery, and fetal outcomes. Odds ratios (ORs) with 95% confidence intervals (CIs) were estimated using a logistic regression model adjusted for age, occupation, marital status, educational level, leisure-time physical activity, smoking habit, body mass index (continuous), number of previous pregnancies, hypertension, diabetes, and hyperlipidemia.

In a multivariable-adjusted analysis (Model 2), each one-unit increment in the MEDI-LITE was associated with lower odds of adverse pregnancy outcomes (OR = 0.94; 95%CI 0.88 to 0.99). Associations with cesarean delivery (OR = 0.96; 95% CI: 0.90–1.01) and stillbirth (OR = 0.94; 95% CI: 0.83–1.06) were in a similar direction, but estimates were imprecise and not conclusive, likely due to the limited number of events (Table 2 and Figure 2). Analyses of individual components of the MEDI-LITE score revealed that, compared to higher consumption, moderate (1–1.5 portions/day) consumption of milk and dairy products was associated with a reduced risk of adverse pregnancy outcomes (OR = 0.58; 95% CI: 0.35 to 0.95) (Table 3). Moderate-to-high intake of fruit (≥1 serving/d) was associated with a lower likelihood of cesarean delivery (OR = 0.65; 95% CI: 0.49–0.87 for moderate intake, and OR = 0.60; 95% CI: 0.40–0.88 for high intake; *p* value for trend = 0.004), and a downward trend with odds of stillbirth was also found (*p* value for trend = 0.038) (Table 4). Consumption of cereals was linked to reduced likelihood of stillbirth (*p* value for trend = 0.049), whereas increasing frequency of olive oil consumption was associated with higher odds of cesarean delivery (*p* value for trend = 0.036; Table 4). Additionally, when examining the individual outcomes (adverse pregnancy outcomes and delivery complications), we observed that higher adherence to the MD was associated with a reduced risk of delivery complications (OR = 0.93; 95% CI: 0.87 to 0.99). Neither adverse pregnancy outcomes nor the category ‘more than one complication’ showed associations with the MD (Supplementary Table S2). Fetal outcomes were excluded from this analysis due to the very small sample size (*n* = 18).

## 4. Discussion

This study found that pregnant women reporting habitual adherence to the MD have lower odds of experiencing adverse pregnancy outcomes, including maternal, fetal, and delivery-related outcomes.

Adverse outcomes during pregnancy warrant careful consideration, as they can have significant long-term implications for both maternal and offspring health. In particular, women diagnosed with gestational diabetes mellitus showed a higher risk of prenatal complications and an increased risk of developing type 2 diabetes in the years following childbirth [22]. Furthermore, children born to mothers with gestational diabetes mellitus are more prone to obesity [23] and have an elevated risk of developing diabetes during early adulthood [24].

Our findings align with a growing body of evidence supporting the protective role of the MD in reducing the risk of adverse pregnancy outcomes. A U.S.-based cohort study [25] reported an inverse association between MD adherence and adverse pregnancy outcomes, and this has been further corroborated by recent systematic reviews. These reviews have consistently shown that adherence to the MD during pregnancy is associated with a lower risk of preeclampsia, gestational diabetes mellitus, excessive gestational weight gain [7,8], metabolic complications in both mothers and their offspring [26], as well as reduced incidence of childbirth complications, urinary tract infections, and impaired fetal growth [27]. Supporting this evidence, an analysis from the Nurses’ Health Study II found that women adhering to healthful dietary patterns, including the MD, the Dietary Approaches to Stop Hypertension (DASH), and diets scoring high on the Healthy Eating Index, had a significantly lower risk of developing gestational diabetes mellitus [28], suggesting that nutrient-rich diets, particularly those high in plant-based foods and fiber, may play a beneficial role in promoting pregnancy-related health.

Observational findings are further reinforced by intervention studies, which have demonstrated reduced risks of developing gestational diabetes mellitus and other adverse maternal–fetal outcomes. This was recently shown in a study involving Spanish women who received dietary guidance based on the MD, supplemented with extra virgin olive oil and pistachios, compared to a control group that was advised to limit fat intake [29]. Again, the ESTEEM trial, designed to estimate the MD’s effects on maternal and fetal outcomes, highlighted that adherence to MD can help limit gestational weight gain and reduce the risk of gestational diabetes mellitus [30]. A favorable role of an MD in preventing pregnancy complications (e.g., gestational diabetes mellitus, preterm birth, and hypertensive disorders of pregnancy) is biologically plausible. This diet is rich in natural sources of antioxidants, mono- and polyunsaturated fats, and phytochemicals that may reduce oxidative stress and systemic inflammation [31], key contributors to endothelial dysfunction and hypertensive disorders of pregnancy [32]. Moreover, MD-driven modulation of glucose homeostasis (through reduced glycemic load and improved insulin action) helps maintain healthy blood pressure and prevent abnormal weight gain [33], both of which contribute to improved maternal and fetal outcomes [34].

Pregnancy itself is a condition in which this adaptation and balance can be easily disrupted. Alterations in this process may result in pregnancy complications, deviations in fetal growth patterns, and an increased risk of preterm birth [35]. Excessive oxidative stress can be harmful and is linked to several pregnancy complications, including preeclampsia and gestational diabetes mellitus, mainly through mechanisms that impair placentation [36]. Also, low-grade inflammation has been linked to several adverse pregnancy outcomes, including reduced chance of live birth and increased risk of pregnancy loss [37].

In our study, an inverse association between MD adherence and the likelihood of cesarean delivery was observed. Although the estimate was consistent in direction with the main findings, the confidence interval was wide and did not allow firm conclusions, likely due to limited statistical power. Potential underlying mechanisms may include better weight management during pregnancy, reduced systemic inflammation, and improved overall metabolic health, all of which can influence the risk of cesarean section [38].

For stillbirth outcomes, the estimated effect size was identical to that observed for the primary adverse pregnancy outcome (OR = 0.94); however, the substantially wider confidence interval, reflecting the low number of events, limited the precision of the estimate and precluded definitive conclusions.

Exploratory analyses of individual components of the MD revealed a potential role of low-to-moderate milk and dairy consumption in reducing the odds of adverse pregnancy outcomes compared with higher intakes. This finding is in line with prior work indicating a higher risk of gestational diabetes mellitus associated with a dietary pattern characterized by high protein and fat from animal sources [39], and this was further confirmed by a recent meta-analysis of prospective cohort studies [40]. Experimental evidence suggests that high-fat diets impair glucose tolerance, an effect accompanied by reduced basal and insulin-stimulated glucose metabolism [41]. In addition, meals rich in animal protein may lead to higher plasma levels of branched-chain amino acids, which have been positively associated with the development of insulin resistance and the onset of diabetes [42]. However, the observed protective association of moderate dairy intake may reflect the beneficial role of nutrients abundant in dairy products, particularly calcium and vitamin D. Calcium is crucial for maternal bone health and for the developing fetal skeleton, teeth, heart, and muscles [43]. Meanwhile, maternal vitamin D has been linked, among others, to appropriate fetal growth and immune function [44].

Moderate-to-high fruit consumption was related to a lower likelihood of cesarean section, and a downward trend was also observed for stillbirth. Fruits are rich in bioactive fibers with prebiotic properties and polyphenols, both of which are known to support maternal health [45]. While no previous study has directly linked fruit intake to mode of delivery, poor fruit consumption has been associated with other adverse pregnancy outcomes, including preterm birth, low birth weight, and stillbirth [46,47].

In addition, increased cereal consumption was associated with a lower likelihood of stillbirth. Cereals, especially whole grains, are key sources of fiber, B vitamins (e.g., folate), iron, and magnesium, which are critical for fetal development and placental health [48]. The high fiber content may also support glycemic control and reduce inflammation [49], helping prevent conditions such as gestational diabetes and preeclampsia, which are linked to higher stillbirth risk [50]. However, given the limited number of stillbirth cases, these findings should be considered exploratory and warrant confirmation in larger studies. Although some food groups of the MD emerged as key contributors, we did not directly examine interactions among its components. Therefore, our findings reflect the role of individual foods rather than the full synergistic effects of the dietary pattern.

Finally, it is worth noting that while olive oil is a key component of the MD and is known for its antioxidant and anti-inflammatory properties, its association with increased cesarean delivery odds may be due to residual confounding or reverse causation, with, e.g., women at higher risk of complications switching to a healthier diet after diagnosis. Moreover, higher olive oil consumption may simply reflect traditional cooking practices that use generous amounts of oil, leading to increased overall energy intake. Excessive energy intake from olive oil could also contribute to gestational weight gain or fetal macrosomia [51], both established factors for cesarean delivery [52].

### Strengths and Limitations

The main strengths of this multi-center study are its reliance on electronic health records, including a substantial number of patients along with detailed clinical, demographic, and laboratory data, and the possibility of following up individuals over time. Also, this study provides a real “big data” perspective, collecting large volumes of data rapidly, without being constrained by a specific study design.

However, our study has several important limitations. First, this is an observational, cross-sectional study, and therefore does not allow for causal inference. This design implies that all associations observed should be interpreted with caution, as temporal relationships between dietary exposure and pregnancy outcomes cannot be firmly established. Nevertheless, reverse causality bias is considered unlikely, as dietary exposure was assessed through a questionnaire on eating habits during the previous year, which could not have been influenced by the occurrence of pregnancy complications or the delivery event. Second, residual confounding cannot be completely excluded despite adjustments for multiple covariates. Third, dietary data were collected using a dietary screener based on self-reported information, which may lead to recall and social desirability bias and to difficulties in assessing portion size. Moreover, other self-reported variables, including lifestyle factors, smoking status, and health metrics such as BMI, may also be subject to reporting inaccuracies, potentially affecting the observed associations.

However, the validated nine-item MEDI-LITE questionnaire, combined with standardized data collection and trained personnel assistance, likely minimized this limitation. Although tools like the MEDI-LITE score are not ideal for detailed dietary assessment, particularly in populations with specific nutritional requirements such as pregnant women, they are straightforward to administer and offer a practical approach for evaluating overall dietary patterns in large-scale studies. Nevertheless, the MEDI-LITE questionnaire does not allow quantification of specific nutrients such as omega-3 fatty acids, antioxidant intake, or total energy intake, which may influence pregnancy outcomes through effects on BMI and metabolic status. Analyses of individual MEDI-LITE components should be interpreted with caution, as they are secondary and potentially underpowered, do not account for the correlations and synergistic effects among dietary factors within the overall Mediterranean pattern, and are therefore susceptible to residual confounding and multiple-testing issues; accordingly, they can only be considered exploratory and hypothesis-generating rather than evidence that specific foods drive the observed associations.

Fourth, the lack of analysis for specific adverse pregnancy outcomes, due to the relatively low number of cases observed, limits the ability to draw definitive conclusions for individual complications. In particular, outcomes such as stillbirths were rare in our cohort, which may have reduced the statistical power to detect significant associations and warrants caution when interpreting these results.

We acknowledge that recruitment occurred during the COVID-19 pandemic and the subsequent lockdown; however, dietary intake was assessed only once for the year preceding enrollment, and potential pandemic-related changes could not be captured.

Fifth, although we adjusted for several covariates in the analyses, unmeasured confounding cannot be completely excluded. Factors such as genetic predispositions, environmental exposures, or other lifestyle habits not captured in our study may have influenced the observed associations. This limitation, combined with the observational and cross-sectional design of the study, reinforces the need for cautious interpretation of the findings. Sixth, our sample included only hospitalized women from Central and Southern Italy. Therefore, these findings may not be generalizable to non-hospitalized pregnant women or to populations with different dietary habits, healthcare systems, and socioeconomic backgrounds.

Furthermore, while our study highlights potential benefits of the MD, it does not account for individual dietary needs or preferences, and thus reflects population-level associations rather than recommendations for individual women.

Women requiring hospitalization may differ in health status or socio-demographic characteristics from the general pregnant population, potentially introducing selection bias. In addition, women hospitalized for day surgery or admitted to intensive care units were excluded, which may have led to underrepresentation of the most severe pregnancy complications and could have affected the observed associations, as hospitalized women may already have a higher baseline risk of pregnancy complications independent of dietary habits.

Finally, although the included and excluded participants did not differ in age, we cannot rule out selection bias due to unmeasured characteristics.

## 5. Conclusions

This study indicates that greater adherence to the MD, as assessed by the MEDI-LITE score, is associated with lower odds of adverse pregnancy outcomes. As this was an observational analysis, and because MEDI-LITE is a validated adherence score rather than a tool to test causal effects on pregnancy outcomes, the findings should be interpreted as associations and do not allow causal inference.

Nevertheless, they support further evaluation, ideally in prospective studies and intervention trials, of strategies to encourage Mediterranean-style dietary patterns during pregnancy, including nutrition education and counseling.

## Figures and Tables

**Table 1:** Characteristics of the pregnant women cohort overall and across levels of adherence to the Mediterranean Diet, as measured by the MEDI-LITE score.

		Adherence to the Mediterranean Diet			
Variables	All	Low (3–9)	Medium (10–11)	High (12–17)	*p*-Value
N of participants (%)	1511	361 (23.9)	685 (45.3)	465 (30.8)	–
MEDI-LITE (mean; min–max)	10.6 (1.8; 3–17)	8.2 (1.0; 3–9)	10.5 (0.5; 10–11)	12.7 (1.0; 12–17)	<0.0001
Age (years; mean, SD)	33.1 (5.2)	32.4 (5.2)	33.0 (5.4)	33.6 (4.9)	0.02
Educational level (%)					0.013
Up to lower school	211 (14.0)	67 (18.6)	94 (13.7)	54 (10.7)	
Upper secondary	766 (50.7)	188 (52.1)	341 (49.8)	237 (50.9)	
Postsecondary education	509 (33.7)	100 (27.7)	240 (35.0)	169 (36.3)	
Missing data	25 (1.6)	6.0 (1.7)	10 (1.5)	9.0 (1.9)	
Occupation (%)					0.21
Manual	356 (23.6)	103 (28.2)	133 (19.4)	120 (25.8)	
Non-manual	534 (35.3)	104 (28.8)	256 (37.4)	174 (37.4)	
Other	577 (38.2)	140 (38.7)	276 (40.3)	161 (34.6)	
Missing data	44 (2.9)	14 (3.9)	20 (2.9)	10 (2.1)	
Marital occupation (%)					0.013
Manual	637 (32.2)	164 (45.4)	290 (42.3)	183 (39.3)	
Non-manual	487 (32.2)	91 (25.2)	235 (34.3)	161 (34.6)	
Other	50 (3.3)	9.0 (2.5)	24 (3.5)	17 (3.7)	
Missing data	337 (22.3)	97 (26.9)	136 (19.8)	104 (22.4)	
Marital status (%)					0.28
Living in a couple	1317 (87.2)	304 (84.2)	600 (87.6)	413 (88.8)	
Separated	28 (1.8)	6.0 (1.7)	18 (2.6)	4.0 (0.9)	
Unmarried	159 (10.5)	50 (13.8)	64 (9.3)	45 (9.7)	
Missing data	7.0 (0.5)	1.0 (0.3)	3.0 (0.4)	3.0 (0.6)	
Body mass index (kg/m^2^; mean, SD) *	25.8 (5.1)	26.0 (5.0)	25.9 (5.2)	25.6 (5.2)	0.60
Smoking status (%)					0.012
Yes	342 (22.6)	108 (29.9)	139 (20.3)	95 (20.4)	
No	988 (65.4)	210 (58.2)	471 (68.8)	307 (66.0)	
Former	180 (11.9)	43 (11.9)	74 (10.8)	63 (13.5)	
Missing data	1.0 (0.1)	0.0	1.0 (0.1)	0.0	
Physically active lifestyle (%)					0.009
Yes	866 (57.3)	200 (55.4)	369 (53.9)	297 (63.9)	
No	620 (41.0)	150 (41.5)	307 (44.8)	163 (35.1)	
Missing data	25 (1.7)	11 (3.0)	9.0 (1.3)	5.0 (1.1)	
Sleep duration (%)					0.27
<4 h	25 (1.7)	6.0 (1.7)	11 (1.6)	8.0 (1.7)	
5–6 h	275 (18.1)	61 (16.9)	125 (18.2)	88 (18.9)	
6–7 h	640 (42.3)	142 (39.3)	309 (45.1)	189 (40.7)	
7–8 h	488 (32.3)	135 (37.4)	205 (29.9)	148 (31.8)	
>8 h	79 (5.2)	16 (4.4)	32 (4.7)	31 (6.7)	
Missing data	5.0 (0.4)	1.0 (0.3)	3.0 (0.4)	1.0 (0.2)	
Hypertension (%)					0.90
Yes	37 (2.4)	8.0 (2.2)	18 (2.6)	11 (2.4)	
No	1469 (97.2)	350 (97.0)	666 (97.2)	453 (97.4)	
Missing data	5.0 (0.3)	3.0 (0.8)	1.0 (0.1)	1.0 (0.2)	
Diabetes (%)					0.24
Yes	9.0 (0.6)	0.0	7.0 (1.0)	2.0 (0.4)	
No	1492 (98.7)	357 (98.9)	673 (98.2)	462 (99.4)	
Missing data	10 (0.7)	4.0 (1.1)	5.0 (0.7)	1.0 (0.2)	
Hyperlipidemia (%)					0.04
Yes	10 (0.7)	4.0 (1.1)	4.0 (0.6)	2.0 (0.4)	
No	1496 (99.0)	353 (97.8)	680 (99.3)	463 (99.6)	
Missing data	5.0 (0.3)	4.0 (1.1)	1.0 (0.1)	0.0	

*p*-Values were obtained using generalized linear models for both continuous and categorical dependent variables, adjusted for age. * Available for 1498 participants.

**Table 2:** Association between adherence to the Mediterranean Diet measured through the MEDI-LITE score with adverse pregnancy outcomes, birth outcomes, and mode of delivery among pregnant women.

	Adherence to the Mediterranean Diet				
	Low (0–9)	Medium (10–11)	High (12–17)		One-Unit Increment in MEDI-LITE
Reference: no adverse pregnancy outcomes	Adverse Pregnancy Outcomes (*n* = 873) in livebirth (*n* = 1428)				
N of cases/*n* of participants	219/341	391/645	263/442	*p*-value for trend	873/1428
Model 1 (OR; 95% CI)	−1-	0.83 (0.64 to 1.10)	0.78 (0.58 to 1.04)	0.10	0.91 (0.88 to 0.99)
Model 2 (OR; 95% CI)	−1-	0.84 (0.64 to 1.12)	0.80 (0.59 to 1.08)	0.16	0.94 (0.88 to 0.99)
Reference: vaginal delivery	Vaginal delivery (*n* = 681) vs. Cesarean section (*n* = 747) in livebirth (*n* = 1428)				
N of cases/*n* of participants	178/341	295/645	223/442	*p*-value for trend	747/1428
Model 1 (OR; 95% CI)	−1-	1.06 (0.81 to 1.38)	0.85 (0.64 to 1.13)	0.21	0.95 (0.89 to 1.00)
Model 2 (OR; 95% CI)	−1-	1.04 (0.79 to 1.27)	0.87 (0.65 to 1.16)	0.30	0.96 (0.90 to 1.01)
Reference: livebirth	Livebirth (*n*= 1428) vs. Stillbirth (*n* = 83)				
N of cases/*n* of participants	20/361	40/685	23/465	*p*-value for trend	83/1511
Model 1 (OR; 95% CI)	−1-	0.99 (0.57 to 1.73)	1.22 (0.65 to 2.26)	0.51	1.07 (0.95 to 1.20)
Model 2 (OR; 95% CI)	−1-	1.10 (0.62 to 1.96)	0.88 (0.47 to 1.65)	0.65	0.94 (0.83 to 1.06)

Data are expressed as Odds Ratios (ORs) with 95% confidence intervals (95% CIs). Model 1 was controlled for age. Model 2 included age, occupation, marital status, educational level, leisure-time physical activity, smoking habit, body mass index (continuous), number of previous pregnancies, hypertension, diabetes, and hyperlipidemia.

**Table 3:** Association between individual dietary components of the MEDI-LITE with adverse pregnancy outcomes among pregnant women from the PLATONE Project cohort.

Individual Components of the MEDI-LITE (Serving/d)	Adverse Pregnancy Outcomes (*n* = 873) in Livebirth (*n* = 1428)		
	N of Cases/*n* of Participants	OR (95%CI)	*p*-Value for Trend
Fruit			
<1	216/386	−1-	0.36
1–1.5	494/821	0.87 (0.65 to 1.16)	
>2	163/269	0.83 (0.55 to 1.24)	
Vegetables			
<1	151/241	−1-	0.87
1–2.5	582/963	0.92 (0.67 to 1.26)	
>2.5	140/224	0.95 (0.59 to 1.53)	
Legumes (serving/week)			
<1	81/130	−1-	0.75
1–2	607/991	1.08 (0.72 to 1.63)	
>2	185/307	0.96 (0.61 to 1.53)	
Cereals			
<1	160/258	−1-	0.88
1–1.5	559/933	0.98 (0.73 to 1.32)	
>1.5	154/237	1.04 (0.70 to 1.55)	
Fish (serving/week)			
<1	137/215	−1-	0.34
1–2.5	597/990	0.85 (0.62 to 1.18)	
>2.5	139/223	0.82 (0.54 to 1.26)	
Meat and processed meat			
>1.5	67/102	−1-	0.13
1–1.5	270/413	1.24 (0.73 to 2.10)	
<1	536/913	0.99 (0.59 to 1.65)	
Milk and dairy products			
>1.5	86/119	−1-	0.37
1–1.5	488/817	0.58 (0.35 to 0.95)	
<1	299/492	0.60 (0.36 to 1.00)	
Alcoholic beverages (A.U./d)			
>2	98/155	−1-	0.59
<1	747/1.224	1.06 (0.70 to 1.60)	
1–2	28/49	0.79 (0.39 to 1.60)	
Olive oil			
Occasionally	25/44	−1-	0.58
Frequently	84/118	1.71 (0.81 to 3.62)	
Regularly	764/1266	1.20 (0.64 to 2.26)	

One alcohol unit (A.U.) is equivalent to one glass of wine (125 mL) or one can of beer (330 mL) Data are expressed as Odds Ratios (ORs) with 95% confidence intervals (95% CI). Analyses included all individual dietary components simultaneously and were controlled for age, occupation, marital status, educational level, leisure-time physical activity, smoking habit, body mass index (continuous), number of previous pregnancies, hypertension, diabetes, and hyperlipidemia.

**Table 4:** Association between individual dietary components of the MEDI-LITE with birth outcomes and mode of delivery among pregnant women from the PLATONE Project cohort.

Individual Components of the MEDI-LITE (Serving/d)	Vaginal Delivery (*n* = 681) vs. Cesarean Section (*n* = 747) in Livebirth (*n* = 1428)			Livebirth (*n* = 1428) vs. Stillbirth (*n* = 83)		
	N of Cases/*n* of Participants	OR (95%CI)	*p*-Value for Trend	N of Cases/*n* of Participants	OR (95%CI)	*p*-Value for Trend
Fruit						
<1	205/338	−1-	0.004	25/363	−1-	0.038
1–1.5	415/821	0.65 (0.49 to 0.87)		47/868	0.62 (0.35 to 1.08)	
>2	127/269	0.60 (0.40 to 0.88)		11/280	0.46 (0.19 to 1.08)	
Vegetables						
<1	138/241	−1-	0.62	11/252	−1-	0.44
1–2.5	500/963	0.89 (0.65 to 1.22)		62/1025	1.80 (0.88 to 3.71)	
>2.5	109/224	0.91 (0.57 to 1.44)		10/234	1.48 (0.39 to 5.56)	
Legumes (serving/week)						
<1	60/130	−1-	0.53	10/140	−1-	0.80
1–2	534/991	1.38 (0.93 to 2.06)		55/1046	0.74 (0.34 to 1.60)	
>2	153/307	1.28 (0.82 to 2.02)		18/325	0.82 (0.34 to 2.00)	
Cereals						
<1	136/258	−1-	0.54	20/278	−1-	0.049
1–1.5	492/933	0.99 (0.74 to 1.32)		53/986	0.69 (0.39 to 1.21)	
>1.5	119/237	0.86 (0.58 to 1.26)		10/247	0.43 (0.19 to 1.01)	
Fish (serving/week)						
<1	113/215	−1-	0.48	10/225	−1-	0.25
1–2.5	527/990	0.95 (0.69 to 1.31)		57/1047	1.26 (0.61 to 2.59)	
>2.5	107/223	0.83 (0.55 to 1.25)		16/239	1.66 (0.68 to 4.05)	
Meat and processed meat						
>1.5	49/102	−1-	0.10	9/111	−1-	0.20
1–1.5	205/413	0.97 (0.59 to 1.60)		24/437	0.63 (0.24 to 1.65)	
<1	493/913	1.17 (0.71 to 1.91)		50/963	0.52 (0.20 to 1.36)	
Milk and dairy products						
>1.5	60/119	−1-	0.60	8/127	−1-	0.88
1–1.5	434/817	1.02 (0.64 to 1.62)		46/863	1.04 (0.33 to 2.76)	
<1	253/492	0.95 (0.60 to 1.52)		29/521	1.00 (0.39 to 2.68)	
Alcoholic beverages (A.U./d)						
>2	83/155	−1-	0.17	8/163	−1-	0.35
<1	642/1224	0.83 (0.56 to 1.24)		69/1293	0.99 (0.41 to 2.42)	
1–2	22/49	0.61 (0.31 to 1.23)		6/55	2.03 (0.60 to 6.89)	
Olive oil						
Occasionally	18/44	−1-	0.036	2/46	−1-	0.13
Frequently	56/118	1.21 (0.58 to 2.52)		3/121	0.43 (0.06 to 2.84)	
Regularly	673/1266	1.60 (0.84 to 3.04)		78/1344	1.40 (0.31 to 6.29)	

One alcohol unit (A.U.) is equivalent to one glass of wine (125 mL) or one can of beer (330 mL).

## Data Availability

The data underlying this article will be shared on reasonable request to the corresponding author. The data are stored in an institutional repository (https://repository.neuromed.it), and access is restricted by the ethical approvals and the legislation of the European Union.

## Supplementary Materials

The following supporting information can be downloaded at https://www.mdpi.com/article/10.3390/nu18050769/s1. Figure S1: Flowchart of the study population; Table S1. Main causes of adverse pregnancy outcomes. Table S2. Association between adherence to the Mediterranean Diet measured through the MEDI-LITE score with adverse pregnancy outcomes and delivery complications among pregnant women.
