# Supplementary material for: Maternal Adherence to the Mediterranean Diet and Adverse Pregnancy Outcomes: Findings from the Multi-Center PLATONE Project
Source: Nutrients. 2026 Feb 26;18(5):769. doi: 10.3390/nu18050769 (PMC12986557; doi:10.3390/nu18050769)
Supplement: Supplementary file 1 [file nutrients-18-00769-s001.zip › nutrients-4078707-supplementary.pdf]

Supplementary file

APPENDIX

Clinical Network Big Data and Personalised Health Project Study Investigators

Coordinator: Licia Iacoviello, MD, PhD, (IRCCS Neuromed, Pozzilli and LUM, Casamassima, Italy), Giovanni de Gaetano (IRCCS Neuromed, Pozzilli, Italy)

Steering Committee: Carmine Malzoni, MD, (Malzoni Research Hospital, Avellino), Paola De Domenico (I.C.M. Agropoli), Giovanni Ricco (Villa del Sole, Salerno), Augusto Di Castelnuovo (Clinica Mediterranea, Napoli)

Recruitment coordinator: Simona Esposito

Neuromed Research Network:

I.R.C.C.S. Neuromed, Pozzilli: Simona Esposito, Sabatino Orlandi, Marialaura Bonaccio, Alessandro Gialluisi, Teresa Panzera, Simona Costanzo

Clinica Malzoni, Avellino: Mario Malzoni, Carmine Malzoni, Elena Bonanno, Maria Bianco

Diagnostica Medica, Avellino: Paola Bruni

Villa del Sole, Salerno: Maria Ceglia, Maria Grazia Caputo, Michelina Contangelo, Maria Rosaria Pandolfi, Pietro Mastandrea, Giovanni Ricco

Clinica Mediterranea, Napoli: Augusto Di Castelnuovo, Francesco Gianfagna, Franco Paradiso, Francesca De Micco

I.C.M., Agropoli: Paola De Domenico, Aniello Formisano, Mariafiorella Tomasino, Clara Di Marco, Moreno Treffiletti.

Biobanking: Amalia De Curtis, Sara Magnacca and Maria Benedetta Donati

Supplementary Figure S1.

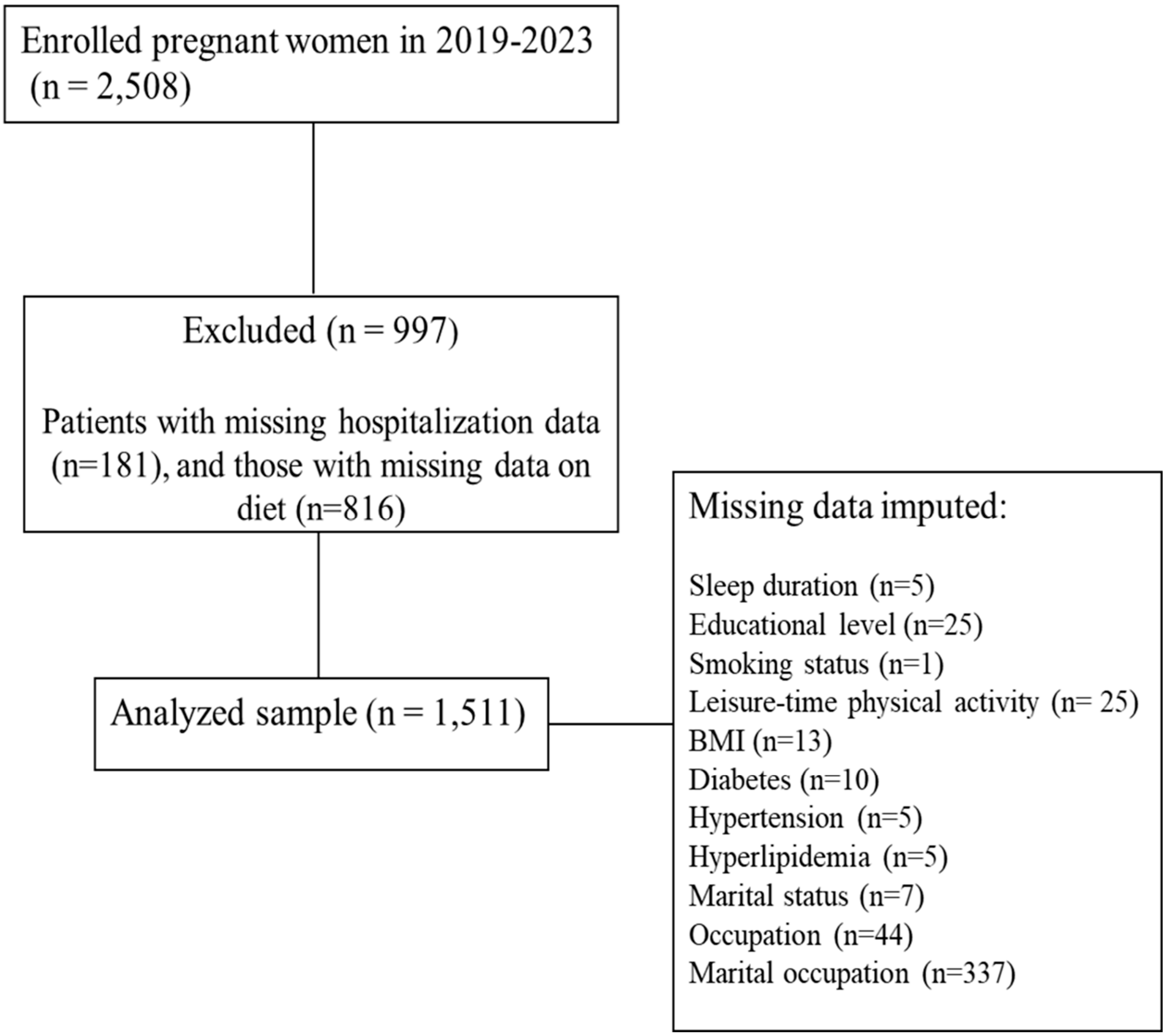

Supplementary Table S1. Main causes of adverse pregnancy outcomes

Adverse pregnancy outcomes	N of cases (%)
Previous cesarean delivery complicating pregnancy, labor, and the puerperium	336 (38.4%)
Ballottable fetal head at term	116 (13.2%)
Prolonged pregnancy	88 (10.1%)
Oligohydramnios	68 (7.8%)
Breech presentation without mention of external cephalic version	41 (4.7%)
Premature rupture of membranes	28 (3.2%)
Secondary uterine inertia, labor, with or without mention of antepartum manifestations	26 (3.0%)
Transverse or oblique presentation	24 (2.8%)
Inadequate fetal development	16 (1.8%)
Other specified abnormality of fetal position or presentation	15 (1.7%)
Impaired glucose tolerance	10 (1.1%)
Delivery with forceps or vacuum extractor, without mention of indication	9.0 (1.1%)
Mild or unspecified preeclampsia	8.0 (0.9%)
Diabetes mellitus, delivery, with or without mention of antepartum condition	7.0 (0.8%)
Polyhydramnios, delivery, with or without mention of antepartum condition	7.0 (0.8%)
Preterm onset of labor, delivery, with or without mention of antepartum condition	7.0 (0.8%)
Prolonged pregnancy, delivery, with or without mention of antepartum condition	5.0 (0.6%)
Previous cesarean delivery complicating pregnancy, labor, and the puerperium, antepartum condition or complication	5.0 (0.6%)
Excessive fetal growth	5.0 (0.6%)
Placenta previa without hemorrhage	4.0 (0.5%)
Fetopelvic disproportion	4.0 (0.5%)
Fetal heart rate or rhythm abnormalities	4.0 (0.5%)
Other and unspecified uterine inertia	4.0 (0.5%)
Placental abruption	3.0 (0.4%)
Decreased fetal movements	3.0 (0.4%)
Liver disorders in pregnancy	2.0 (0.2%)
Other specified or suspected liver disorders, not elsewhere classified	2.0 (0.2%)
Cesarean delivery, without mention of indication, with or without mention of antepartum condition or complication	2.0 (0.2%)
Other antepartum hemorrhage, delivery, with or without mention of antepartum condition or complication	1.0 (0.1%)
Hypertension secondary to renal disease complicating pregnancy, labor, and the puerperium, delivery, with or without mention of antepartum condition or complication	1.0 (0.1%)

Other persistent hypertension complicating pregnancy, labor, and the puerperium, delivery, with or without mention of antepartum condition or complication	1.0 (0.1%)
Transient hypertension of pregnancy, delivery, with or without mention of antepartum condition or complication	1.0 (0.1%)
Pre-eclampsia or eclampsia superimposed on pre-existing hypertension, delivery, with or without mention of antepartum condition or complication	1.0 (0.1%)
Prolonged pregnancy, antepartum condition or complication	1.0 (0.1%)
Woman with habitual abortion, delivery, with or without mention of antepartum condition or complication	1.0 (0.1%)
Liver disorders in pregnancy, antepartum condition or complication	1.0 (0.1%)
Obesity complicating pregnancy, labor, and the puerperium, delivery, with or without mention of antepartum condition or complication	1.0 (0.1%)
Coagulation defects complicating pregnancy, labor, and the puerperium, delivery, with or without mention of antepartum condition or complication	1.0 (0.1%)
Twin pregnancy, antepartum condition or complication	1.0 (0.1%)
Unstable lie, delivery, with or without mention of antepartum condition or complication	1.0 (0.1%)
Breech presentation without mention of version, antepartum condition or complication	1.0 (0.1%)
Unspecified abnormality of fetal presentation or position, delivery, with or without mention of antepartum condition or complication	1.0 (0.1%)
Other and unspecified abnormalities of pelvic organs or soft tissues	1.0 (0.1%)
Unspecified fetal abnormality, delivery, with or without mention of antepartum condition or complication	1.0 (0.1%)
Fetal distress, delivery, with or without mention of antepartum condition or complication	1.0 (0.1%)
Excessive fetal growth, antepartum condition or complication	1.0 (0.1%)
Other specified fetal and placental problems, delivery, with or without mention of antepartum condition or complication	1.0 (0.1%)
Delayed delivery after spontaneous or unspecified rupture of membranes, delivery, with or without mention of antepartum condition or complication	1.0 (0.1%)
Failed medical or unspecified induction of labor, delivery, with or without mention of antepartum condition or complication	1.0 (0.1%)
Bony pelvic obstruction during labor, delivery, with or without mention of antepartum condition or complication	1.0 (0.1%)
Prolonged second stage of labor, delivery, with or without mention of antepartum condition or complication	1.0 (0.1%)
Other complications of umbilical cord, delivery, with or without mention of antepartum condition or complication	1.0 (0.1%)

Delayed and secondary postpartum hemorrhage, delivery, with or without mention of antepartum condition or complication	1.0 (0.1%)
Other complications of labor and delivery, delivery, with or without mention of antepartum condition or complication	1.0 (0.1%)

Supplementary Table S2. Association between adherence to the Mediterranean Diet measured through the MEDI-LITE score with adverse pregnancy outcomes and delivery complications among pregnant women.

	Adherence to the Mediterranean Diet			
	**Low (0-9)**	**Medium (10-11)**	**High (12-17)**	**1-unit increment in MEDI-LITE**
**Adverse Pregnancy Outcomes* (n=63) vs. no complication (n=552)**				
N of cases/n of participants	17/138	26/279	20/198	63/615
(OR; 95%CI)	-1-	0.73 (0.38 to 1.42)	0.77 (0.38 to 1.57)	0.93 (0.81 to 1.08)
**Delivery complication* (n=635) vs. vs. no complication (n=552)**				
N of cases/n of participants	166/287	283/536	186/364	635/1187
(OR; 95%CI)	-1-	0.79 (0.59 to 1.06)	0.74 (0.54 to 1.03)	0.93 (0.87 to 0.99)
**More than one complication (n=160) vs. no complication (n=552)**				
N of cases/n of participants	35/156	74/327	51/229	160/712
(OR; 95%CI)	-1-	0.99 (0.62 to 1.57)	0.96 (0.58 to 1.57)	0.97 (0.88 to 1.07)

*As the only complication. Data are expressed as Odds Ratios (ORs) with 95% confidence intervals (95%CIs), in the model were included age, leisure-time physical activity, body mass index (continuous) and hypertension.
